# Anti-Proliferative and Pro-Apoptotic Effects of Short-Term Inhibition of Telomerase In Vivo and in Human Malignant B Cells Xenografted in Zebrafish

**DOI:** 10.3390/cancers12082052

**Published:** 2020-07-25

**Authors:** Silvia Giunco, Manuela Zangrossi, Francesca Dal Pozzolo, Andrea Celeghin, Giovanni Ballin, Maria Raffaella Petrara, Aamir Amin, Francesco Argenton, Miguel Godinho Ferreira, Anita De Rossi

**Affiliations:** 1 Oncology and Immunology Section, Department of Surgery, Oncology and Gastroenterology, University of Padova, 35128 Padova, Italy; manuela.zangrossi.btm@gmail.com (M.Z.); andreaceleghin@gmail.com (A.C.); ballin.giovanni@gmail.com (G.B.); raffaella.petrara@unipd.it (M.R.P.); aamir.amin@studenti.unipd.it (A.A.); anita.derossi@unipd.it (A.D.R.); 2 Immunology and Diagnostic Molecular Oncology Unit, Veneto Institute of Oncology IOV–IRCCS, 35128 Padova, Italy; francesca.dalpozzolo08@gmail.com; 3 Department of Biology, University of Padova, 35128 Padova, Italy; francesco.argenton@unipd.it; 4 Institute for Research on Cancer and Aging of Nice (IRCAN), UMR7284 U1081 UNS, Université Côte d’Azur, 06107 Nice CEDEX 2, France; Miguel-Godinho.FERREIRA@unice.fr

**Keywords:** TERT/telomerase, B-cell malignancies, new therapeutic approach, zebrafish, xenograft

## Abstract

Besides its canonical role in stabilizing telomeres, telomerase reverse transcriptase (TERT) may promote tumor growth/progression through extra-telomeric functions. Our previous in vitro studies demonstrated that short-term TERT inhibition by BIBR1532 (BIBR), an inhibitor of TERT catalytic activity, negatively impacts cell proliferation and viability via telomeres’ length-independent mechanism. Here we evaluate the anti-proliferative and pro-apoptotic effects of short-term telomerase inhibition in vivo in wild-type (wt) and *tert* mutant (*tert^hu3430/hu3430^*; *tert−/−*) zebrafish embryos, and in malignant human B cells xenografted in casper zebrafish embryos. Short-term Tert inhibition by BIBR in wt embryos reduced cell proliferation, induced an accumulation of cells in S-phase and ultimately led to apoptosis associated with the activation of DNA damage response; all these effects were unrelated to telomere shortening/dysfunction. BIBR treatment showed no effects in *tert−/−* embryos. Xenografted untreated malignant B cells proliferated in zebrafish embryos, while BIBR pretreated cells constantly decreased and were significantly less than those in the controls from 24 to up to 72 h after xenotransplantation. Additionally, xenografted tumor cells, treated with BIBR prior- or post-transplantation, displayed a significant higher apoptotic rate compared to untreated control cells. In conclusion, our data demonstrate that short-term telomerase inhibition impairs proliferation and viability in vivo and in human malignant B cells xenografted in zebrafish, thus supporting therapeutic applications of TERT inhibitors in human malignancies.

## 1. Introduction

Maintenance of telomeres’ length, required for the unlimited cell proliferation displayed by cancer cells, is provided by telomerase, a ribonucleoprotein complex containing a specialized reverse transcriptase, encoded by the telomerase reverse transcriptase (*TERT*) gene, which uses an internal RNA template (TR) to maintain telomeres’ length, thus playing a critical role in tumor formation and progression [1]. Expression of TERT is restricted to stem cells, and is usually repressed in normal somatic cells. It may be expressed at low levels in normal hematopoietic cells according to their state of differentiation/activation [2]. In contrast, TERT is expressed in the vast majority of tumors [1].

Since the replicative potential of cancer cells is mainly attributable to the inappropriate reactivation of TERT, targeting telomerase may be a promising anticancer strategy, but it is apparently restricted to tumors with short telomeres. However, growing evidence suggests that, besides its canonical role in stabilizing telomeres, TERT may contribute to carcinogenesis also via telomere length-independent mechanisms, including enhancement of cellular proliferation kinetics, modulation of DNA damage responses (DDR), resistance to apoptosis, inflammation, invasion and metastasis through modulation of signal transduction pathways, gene expression regulation, and mitochondrial function [3,4,5,6,7,8].

BIBR1532 (BIBR) is a noncompetitive non-nucleoside small molecule that selectively inhibits telomerase activity [9,10,11,12,13,14,15]. From crystal structural data, it has been advanced that BIBR binds to a hydrophobic pocket, conserved across species, on the superficial region of TERT, thus interfering with telomerase assembly and stability [14]. We have previously demonstrated that short-term TERT inhibition in vitro by BIBR leads to cell cycle arrest in S-phase and induces apoptosis on both Epstein Barr Virus EBV-immortalized lymphoblastoid cell lines (LCLs) and Burkitt’s lymphoma (BL) cells [15]. These effects occur within 72 h of drug exposure and are associated with activation of the DDR, highlighted by increased levels of phosphorylated H2A histone family, member X (γH2AX) and activation of ataxia telangiectasia mutated (ATM) and ataxia telangiectasia and Rad3 related (ATR) pathways. Analyses of the mean and range of telomeres’ lengths and telomere dysfunction-induced foci (TIF) indicated that DDR and subsequent cell apoptosis induced by short-term inhibition of TERT are not associated with telomere shortening [15]. Importantly, TERT inhibition by BIBR enhanced the pro-apoptotic and anti-proliferative effects of chemotherapeutic agents [15]. All together, these in vitro results support the concept that telomerase inhibition could be a useful therapeutic strategy to counteract tumor cell survival and replication, regardless of its effects on telomere length. These promising results need to be validated in an animal model to evaluate in vivo the potential therapeutic effects of TERT inhibition as anticancer therapy.

Danio rerio, known as zebrafish, has emerged as a useful model for studying several areas of cancer research [16], including the characterization of the non-canonical functions of Tert [17]. Zebrafish Tert is a 126 kD protein composed of 1091 amino acids; it displays 36% whole sequence similarity with human TERT, but functional domains of zebrafish telomerase (N-terminal domain, TR binding site, retro-transcriptase motif) are highly similar to their human counterparts [17,18]. Zebrafish TR is also quite conserved, both in structure and function, between zebrafish and human [19]. As observed in humans, telomerase expression decreases over time in zebrafish; indeed, zebrafish embryos show high *tert* mRNA levels from 9 to 24 h post-fertilization (hpf) and Tert protein expression is maintained at high levels up to 72 hpf, beyond which it begins to decrease [17]. The availability of *tert^hu3430/hu3430^* homozygous mutant (*tert−/−*) zebrafish [20,21] as negative control supports the use of this vertebrate model to test the effects of telomerase inhibitors. Moreover, the telomere length (15–20 kb) of zebrafish is relatively similar to the human one (15–20 kb) and shows progressive shortening with age [18]. 

The zebrafish embryo xenograft model has been employed for several cancers, including leukemia [22], as a first step in a preclinical pipeline to screen for antineoplastic drugs before moving on to the more expensive and time-consuming animal system and ultimately human trials [23,24,25]. In addition, xenotransplantation in zebrafish embryos does not require immunosuppression since at early embryonic stages the adaptive immune response has not yet developed [26]. Thus, the high conservation of telomere and telomerase biology between zebrafish and humans, together with the possibility to explore the chemotherapy response in xenotransplanted tumor cells, makes this animal an appropriate model to study the in vivo effects of telomerase inhibition as an anticancer strategy.

In this study, we evaluated the effects of short-term Tert inhibition on cell proliferation and viability in vivo in zebrafish embryos. In addition, we explored the antineoplastic effects of TERT inhibition in malignant human B cells xenografted in zebrafish embryos.

## 2. Results

### 2.1. Short-Term Tert Inhibition by BIBR Reduced Cell Proliferation and Induced an Accumulation of Cells in S-Phase

To determine the effects of short-term Tert inhibition by BIBR in vivo, we treated wild-type (wt) zebrafish embryos at the stage of 12 hpf with serial dilutions of BIBR (Selleckchem, Houston, TX, USA) (0.5, 2 and 4 μM of BIBR dissolved in DMSO), or with DMSO alone as control, and analyzed samples after 12 h of treatment, i.e., at 24 hpf, when then Tert expression was high in zebrafish embryos [17]. At a concentration of 0.5 μM, BIBR did not show any significant effect on wt embryos, whereas at 4 μM it caused almost 50% of embryonic lethality; 2 μM BIBR induced an embryo death rate of 14 ± 0.021% in wt, but showed no effect on mutant *tert−/−* zebrafish (Figure S1). Therefore, 2 μM BIBR concentration was employed for the subsequent experiments.

BIBR has been demonstrated to reduce telomerase activity in several human cancer cell lines [9,10,11,12,13,14,15], but no data are available on zebrafish. Therefore, we tested the effect of BIBR on telomerase activity in zebrafish embryos. As shown in Figure S2, 12 h of treatment with 2 µM BIBR induced a 75.7 ± 8.4% decrease in the level of telomerase activity compared to that of the control (*p* < 0.001). As expected, no evidence of telomerase activity was observed in protein extracts from *tert−/−* zebrafish embryos (Figure S2).

**Figure 1:**
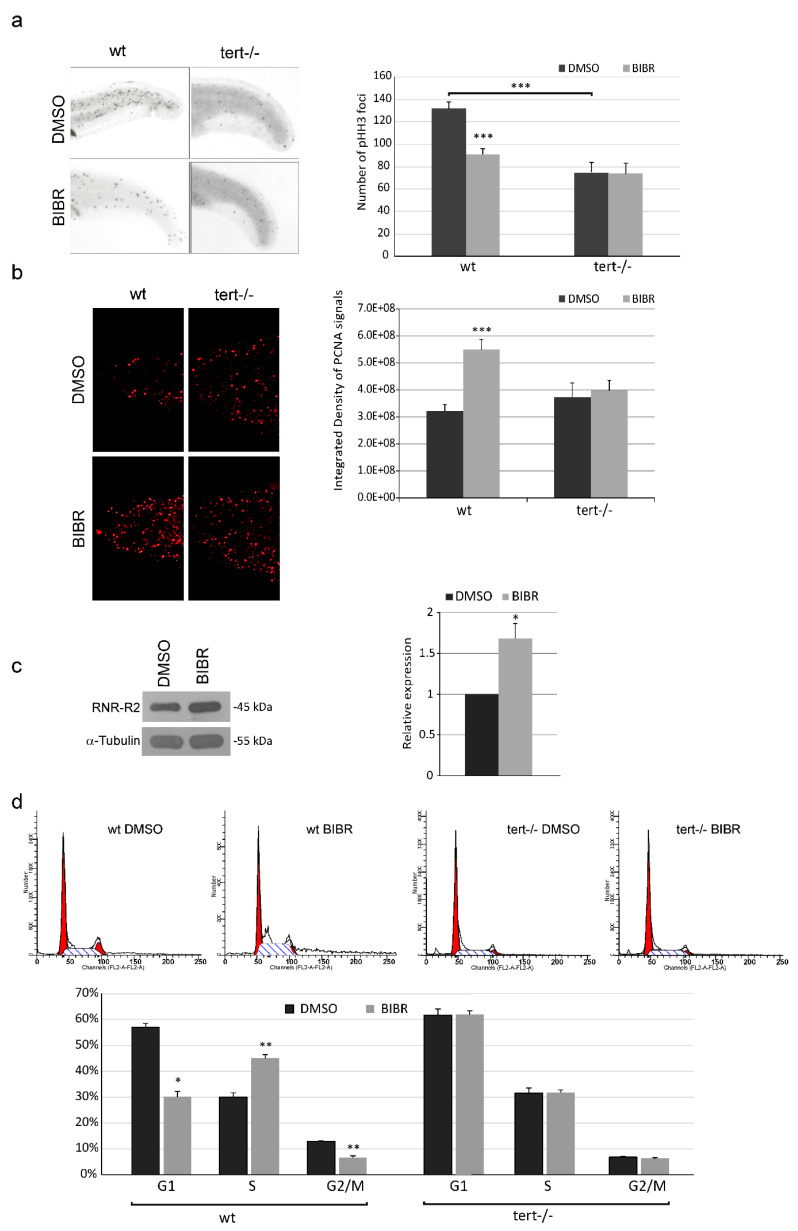
Anti-proliferative effect of short-term telomerase reverse transcriptase (Tert) inhibition by BIBR1532 (BIBR) in zebrafish embryos. (**a**) Representative immunohistochemistry pattern of phosphohistone H3 (pHH3) foci in wt and *tert−/−* embryos treated with BIBR or DMSO, from 12 to 24 hpf. The graph on the right shows the means and standard deviation (SD) (bar) of pHH3 foci in 20 embryos per group. The quantification analysis of pHH3 foci counted in the caudal portion, from the apex of the tail to the anus, was made by ImageJ software. (**b**) Representative immunofluorescence pattern of proliferating cell nuclear antigen (PCNA) in wt and *tert−/−* embryos treated with BIBR or DMSO. The graph on the right shows the relative means and SD (bar) of PCNA expression in 20 embryos per group. The quantification analysis of PCNA expression, as integrated density values, in the caudal portion was made by ImageJ software. (**c**) Representative R2 subunit of ribonucleotide reductase (RNR-R2) protein (45 kD) level, detected by specific antibody, and normalized on tubulin content. The graph on the right shows densitometry analysis in arbitrary units performed with ImageJ software. Data represent the mean and SD (bar) from two separate experiments with value of 1 assigned to DMSO-treated controls (relative expression). (**d**) Representative panels of cell cycle profile analyzed by flow cytometry in propidium iodide (PI)-stained cells from dissociated wt and *tert−/−* embryos treated with BIBR or DMSO. Percentages of cells in G1, S and G2/M-phase are shown in the graph below. A significant difference between values in BIBR-treated embryos versus DMSO-treated embryos is shown: * *p* < 0.05, ** *p* < 0.01, *** *p* < 0.001.

To assess the in vivo modulatory effect of Tert inhibition on cell proliferation, we evaluated the expression of phosphohistone H3 (pHH3), a mitosis molecular marker, in short-term treated zebrafish embryos. Twelve hours of BIBR treatment reduced the proliferation rate in wt embryos, as indicated by the significant decrease in the mitotic foci, counted in the caudal portion, from the apex of the tail to the anus, compared to those observed in control DMSO-treated embryos (91 ± 5 vs. 132 ± 6 foci, respectively; *p* < 0.001) (Figure 1a). Notably, there was a significant difference in the basal pHH3 expression between wt and *tert−/−* embryos (132 ± 6 in wt vs. 75 ± 9 in *tert−/−* foci; *p* < 0.001), but BIBR treatment in *tert−/−* embryos did not affect the number of mitotic foci compared to controls (74 ± 9 vs. 75 ± 9, respectively) (Figure 1a).

To evaluate cell cycle profile upon short-term Tert inhibition, we measured the level of two S-phase specific markers, i.e., proliferating cell nuclear antigen (PCNA) and R2 subunit of ribonucleotide reductase (RNR-R2). Immunofluorescence analyses highlighted a significant increase in PCNA in BIBR-treated wt embryos compared to controls (5.48 ± 0.38 vs. 3.22 ± 0.24 integrated density values/10E8, respectively; *p* < 0.001). Conversely, BIBR treatment showed no effect on PCNA expression in *tert−/−* embryos compared to controls (Figure 1b). Consistently, the protein levels of RNR-R2 confirmed the accumulation of cells in S-phase, with a 1.68 ± 0.19 fold increased expression in BIBR-treated wt embryos compared to controls (*p* = 0.034) (Figure 1c). To further investigate cell cycle profile, we also analyzed by flow cytometry propidium iodide (PI) stained cells obtained from dissociated embryos. According to the aforementioned results, wt embryos treated with BIBR showed cell cycle alterations, with decreased cells in the G1- and G2/M-phase and a significant accumulation of cells in the S-phase (Figure 1d). Instead, BIBR treatment did not affect the cell cycle profile of telomerase mutant *tert−/−* embryos (Figure 1d). These findings demonstrate that short-term telomerase inhibition by BIBR in vivo impairs proliferation by promoting selective accumulation of cells in the S-phase.

### 2.2. Short-Term Tert Inhibition by BIBR Induces Apoptosis and Activates DDR

**Figure 2:**
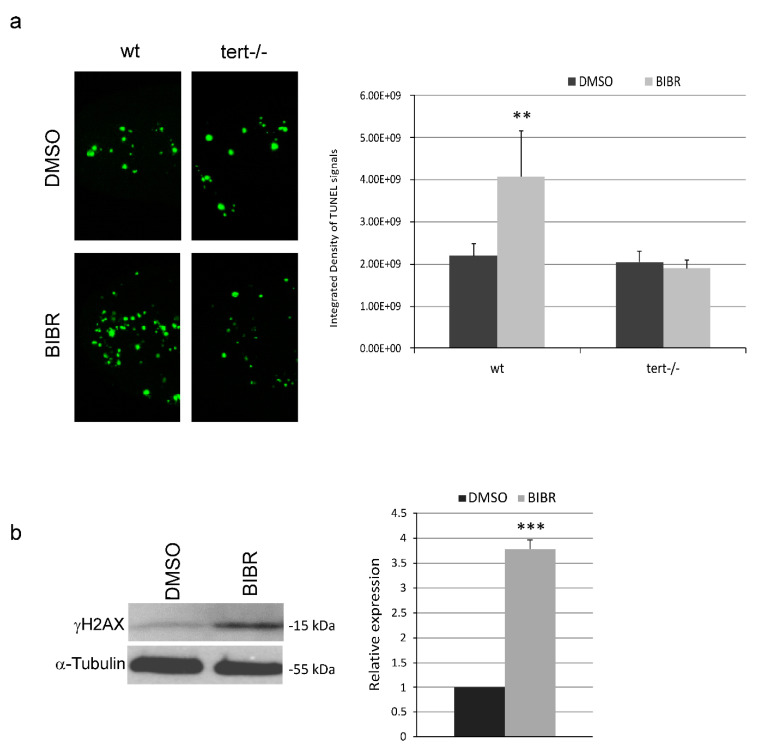
Tert inhibition in zebrafish embryos induces apoptosis and activates DNA damage responses (DDR). (**a**) Representative images of terminal deoxynucleotidyl transferase dUTP nick end labeling (TUNEL) assay in wt and *tert−/−* embryos treated with BIBR or DMSO from 12 to 24 hpf. The quantification analysis of apoptotic foci in the caudal portion, as integrated density values, was made by ImageJ software. The graph on the right shows the relative means and SD (bar) of apoptotic foci in 20 embryos per group. (**b**) Representative western blot analyses of phosphorylated H2A histone family, member X (γH2AX) protein (15 kDa) level, detected by specific antibody, and normalized on tubulin content. The graph on the right shows densitometry analysis in arbitrary units performed with ImageJ software. Data represent the mean and SD (bar) from three separated experiments with value of 1 assigned to DMSO-treated controls (relative expression). A significant difference between values in BIBR-treated embryos versus DMSO-treated embryos is shown: ** *p* < 0.01, *** *p* < 0.001.

To evaluate whether the decrease in proliferation rate was accompanied by cell death, we analyzed the number of apoptotic foci, detected by terminal deoxynucleotidyl transferase dUTP nick end labeling (TUNEL) assay, in zebrafish embryos short-term treated with BIBR. We observed that 12 h of BIBR treatment in wt embryos induced a significant increase in the number of apoptotic foci compared to controls (4.08 ± 1.09 vs. 2.19 ± 0.29 integrated density values/10E9, respectively; *p* = 0.007), while the treatment did not induce apoptosis in *tert−/−* embryos (Figure 2a). Thus, short-term Tert inhibition by BIBR induces apoptosis in vivo.

An important player in inducing apoptosis is the activation of DDR. To assess this possibility we analyzed the expression of γH2AX, a marker of DNA damage, in short-term BIBR-treated wt embryos. As shown in Figure 2b, 12 h of BIBR treatment induced 3.78 ± 0.22 fold increase in the level of γH2AX, compared to controls (*p* < 0.001), thus supporting the concept of apoptosis driven by DNA damage (Figure 2b).

### 2.3. Short-Term Tert Inhibition Did not Affect Telomeres

**Figure 3:**
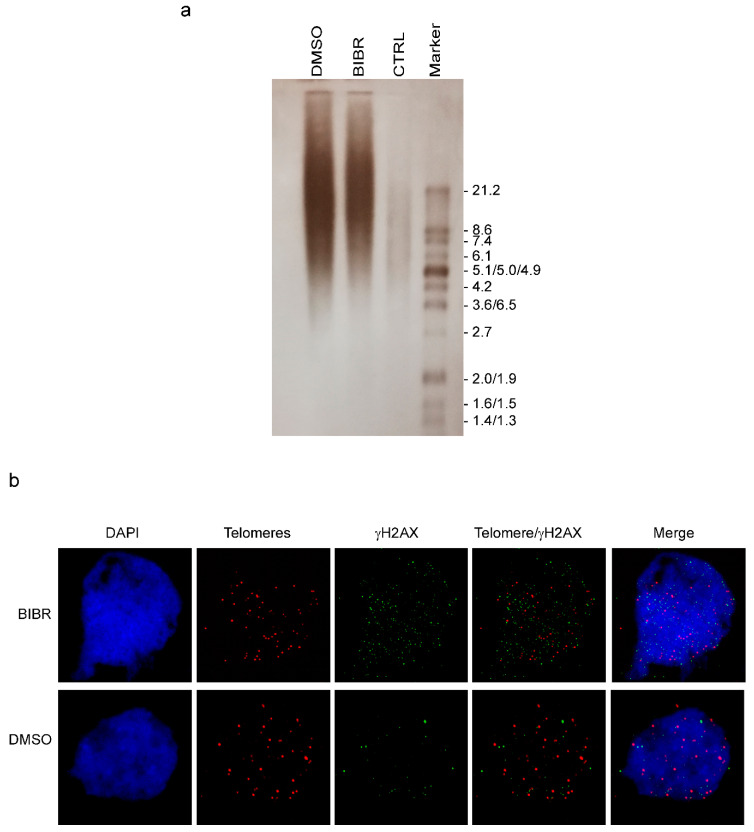
Short-term Tert inhibition does not affect telomeres. (**a**) DNA from wt embryos treated with BIBR or DMSO for 36 h, from 12 to 48 hpf, was employed to visualize the range of telomere length assessed by terminal restriction fragment (TRF) analyses. A panel from one representative experiment is shown. CTRL (control) DNA from TeloTAGGG kit. (**b**) Telomere dysfunction-induced foci (TIF) analysis. Representative micrographs showing the combined telomere fluorescence in situ hybridization (FISH)/γH2AX immunofluorescence of wt zebrafish cells from embryos treated with BIBR or DMSO, from 12 to 24 hpf. From the left: 4′,6-diamidino-2-phenylindole (DAPI) (nuclear marker, blue), telomere probe (red), γH2AX (DNA damage marker, green), combined telomere/γH2AX, and the merged image. Magnification: 100×.

Given that telomere erosion or telomere dysfunction lead to the activation of DDR, we evaluated the involvement of telomeres in the effects observed after the short-term inhibition of Tert in wt zebrafish embryos. We analyzed the telomere length of BIBR-treated wt embryos by terminal restriction fragment (TRF) analysis, which allows us to visualize the range of telomere length. As previously shown by Imamura et al. [17], at 12–48 hpf, we observed that the mean zebrafish wt telomere length is about 15–20 kb (Figure 3a). Interestingly, the TRF lengths were found to be unchanged in the embryos treated for 36 h with 2 µM of BIBR (Figure 3a). This finding was confirmed by evaluating the relative telomere length by quantitative real time PCR (Figure S3). As expected telomeres’ lengths in *tert−/−* were shorter than in wt embryos [20], and they were unaffected by BIBR treatment (Figure S3)**.** Thus, short-term inhibition of Tert in wt zebrafish embryos is not sufficient to elicit detectable telomere shortening.

Moreover, we investigated whether the DNA damage induced by the treatment was specifically located at telomeres, which would indicate the participation of telomere dysfunction in the effects observed after short-term Tert inhibition. At 24 hpf, after 12 h of BIBR exposure, telomere dysfunction induced foci (TIF) assay showed that, in BIBR treated embryos, DNA damage foci, highlighted by γH2AX signals, did not specifically co-localize with the telomere probes, indicating randomly distributed DNA damage throughout the genome (Figure 3b). The percentage of TIF-positive cells containing four or more telomere-specific damage signals [27] was lower than 3% in both DMSO and BIBR treated embryos.

### 2.4. Anti-Proliferative and Pro-Apoptotic Effects of Short-Term TERT Inhibition in Malignant Human B Cells Xenografted in Zebrafish

**Figure 4:**
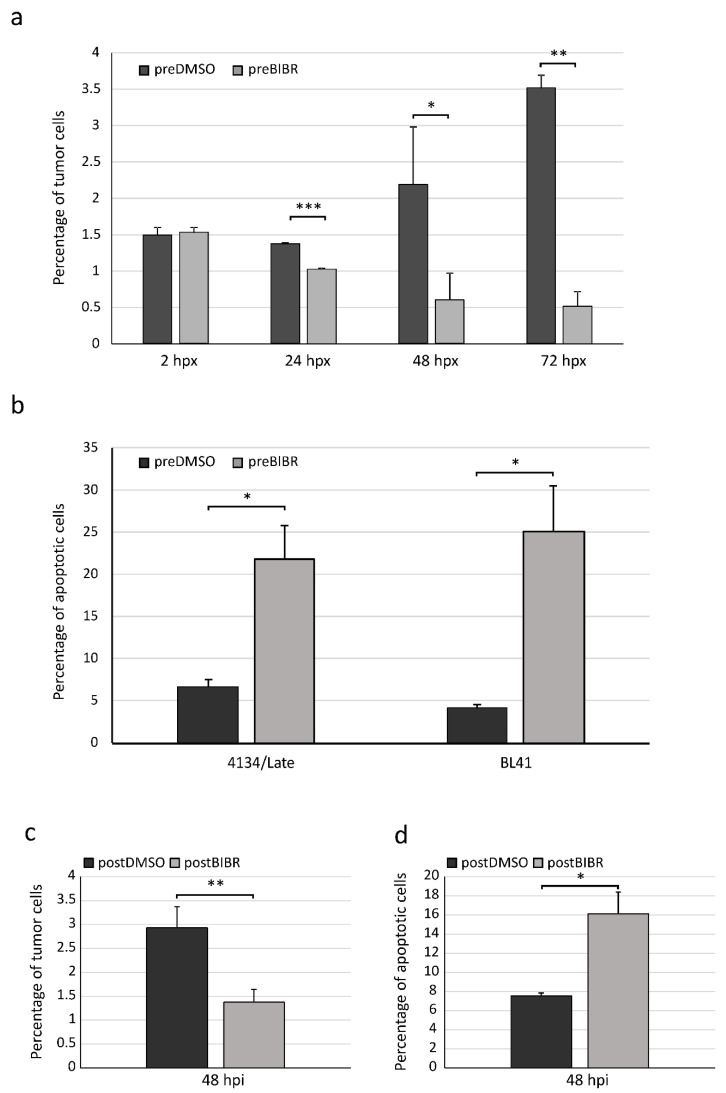
Effects of BIBR on proliferation and viability of tumor cells xenografted in zebrafish embryos. (**a** and **b**) Xenografted fluorescent tumor cells, pretreated with BIBR (preBIBR) or DMSO (preDMSO), were detected by flow cytometry in enzymatically dissociated embryos. (**a**) Percentage of 4134/Late cells in zebrafish embryos according to time post-xenograft (hpx). Values are means and SD (bar) of three separate experiments of 10 embryos per group. (**b**) Cell suspensions from enzymatically dissociated embryos were processed by TUNEL assay for the detection of apoptotic cells. Histograms represent the percentage of tumor apoptotic cells (4134/Late and BL41) at 72 hpx. Values are means and SD (bar) of two separate experiments of 10 embryos per group. (**c** and **d**) Twenty-four hours after transplantation, untreated 4134/Late cells were injected with BIBR (postBIBR) or DMSO (postDMSO). Forty-eight hours after drug injection (hpi), fluorescent tumor cells were analyzed by flow cytometry in enzymatically dissociated embryos for proliferation (**c**) and apoptosis (**d**). * *p* < 0.05, ** *p* < 0.01 and *** *p* < 0.001.

In order to find out whether the anti-proliferative and pro-apoptotic effects of short-term TERT inhibition observed in in vitro models were maintained in in vivo context, we evaluated proliferation and viability of malignant human B cells xenografted in zebrafish. To this aim, we employed LCL 4134/Late and BL41 cells, which are well characterized in vitro models of post-transplant lymphoproliferative disorders and Burkitt’s lymphoma, respectively [15]. Cells were pre-treated for 16 h with 30 µM of BIBR or DMSO as control. Thirty µM of BIBR treatment has proven to be effective in halting viability and proliferation in these cell lines without affecting telomerase-negative cell lines, employed as controls [15]. Subsequently, pre-treated cells were stained with the vital cell tracker red fluorescent chloromethylbenzamido derivative of octadecylindocarbocyanine (CM-DiI) (Invitrogen, Life Technologies, Carlsbad, CA, USA) followed by microinjection into yolk sac of 72 hpf transparent casper zebrafish embryos. Two hours post-xenotransplantation (hpx), successfully xenografted embryos with similar tumor fluorescent intensity were transferred to 32 °C until the end of experiments. The percentage of tumor cells in engrafted embryos was determined at 2, 24, 48 and 72 hpx by enzymatically dissociating 10 embryos per group to obtain single cell suspension for flow cytometry analysis (see the Materials and Methods section for details). To discriminate between fish and human malignant B cells, the fluorescence intensity signal of the CM-DiI fluorochrome was detected as shown in the dot plot of Figure S4a. In each experiment, non-xenografted dissociated embryos were also analyzed to set the experiment threshold (Figure S4a). At 2 hpx the percentage of 4134/Late fluorescent tumor cells was similar between the embryos xenografted with DMSO- or BIBR-pretreated cells (1.49 ± 0.11% vs. 1.53 ± 0.07%, respectively; *p* = 0.623), confirming that an equal number of cells had been injected in the two groups. At 24 hpx the percentage of tumor cells remained stable in embryos injected with DMSO-pretreated cells, while significantly decreased in those injected with BIBR-pretreated cells (1.37 ± 0.001% vs. 1.03 ± 0.01%; *p* = 0.001). At 48 hpx the percentage of tumor cells in embryos xenografted with 4134/Late pretreated with DMSO was significantly higher than those in embryos xenografted with BIBR-pretreated cells (2.18 ± 0.8% vs. 0.60 ± 0.37%; *p* = 0.011). Similarly, at 72 hpx DMSO-pretreated cells proliferated up to 3.51%, whereas the percentage of cells pretreated with BIBR was significantly lower (0.52%; *p* = 0.004) (Figure 4a). These results reflect our previous in vitro data on anti-proliferative effect of BIBR treatment in these cell lines [15].

Xenotransplantation of BL41 cells pretreated with BIBR or DMSO showed a behavior similar to that of 4134/Late cells, displaying higher percentage of DMSO pretreated cells compared to BIBR pretreated ones at each considered time point (Figure S5).

We analyzed the percentage of apoptotic cells xenografted in zebrafish at 72 hpx, when the percentage of cells pre-treated with DMSO or BIBR in dissociated embryos showed the largest difference. Apoptotic rate was detected by TUNEL assay positivity in enzymatically dissociated embryos by flow cytometry selecting CM-DiI fluorescent population (Figure S4b). Figure 4b shows that the reduced percentage of xenografted cells pretreated with BIBR was associated with increased apoptosis compared with those pretreated with DMSO in both 4134/Late and BL41 cells.

To test whether local injection of BIBR was also effective in halting proliferation and viability, we directly inoculated the drug into the untreated 4134/Late xenografted cells. To this purpose, 24 h after transplantation, tumor cells were injected with a solution containing 30 µM of BIBR or DMSO as control (see the Materials and Methods section for details). Similarly to the pre-treatment approach, at 48 h post-injection (hpi), the percentage of tumor cells in embryos injected with DMSO was significantly higher than those in embryos injected with BIBR (2.93 ± 0.44% vs. 1.38 ± 0.27%; *p* = 0.006) (Figure 4c). Consistently, TUNEL assay reflected the pro-apoptotic effect observed in the pre-treatment approach, showing a higher percentage of apoptotic tumor cells in BIBR compared to DMSO-injected embryos (16.11 ± 2.26% vs. 7.53 ± 0.31% *p* = 0.034) (Figure 4d).

## 3. Discussion

Telomerase inhibitors remain an attractive approach to target cancer cells, given the specificity of TERT expression in tumor cells [1]. Telomerase inhibition has already been exploited as an anticancer strategy, since it reduces the proliferative potential of cancer cells after continuous cell divisions within the tumor; indeed, several classes of telomerase inhibitors have been developed, most of which specifically affect telomere maintenance [28]. However, the time to antineoplastic effectiveness of telomerase inhibitors depends on the original length of the telomeres of the cancer cells and, apparently, these agents are effective in halting tumor growth only after the cancer cells have shortened their telomeres. The evidence of extra-telomeric functions of TERT in cellular kinetics and resistance to apoptosis strongly supports the potential telomere length-independent therapeutic effects of TERT inhibition.

This study demonstrates that short-term inhibition of Tert in 24 hpf wt zebrafish embryos reduces cell proliferation, induces an accumulation of cells in S-phase and ultimately leads to apoptosis; these effects are associated with the activation of DDR. Interestingly, as indicated by the analysis of mitotic foci, inhibition of Tert reduced the proliferation rate at the same level observed in *tert−/−* embryos, indicating that the lack of telomerase impairs proliferation at this stage. The contrast between the modest effect seen on *tert−/−* zebrafish and the more severe consequences of Tert inhibition in wt embryos, suggests that Tert function(s) is partially compensated in zebrafish *tert−/−*. While alternative pathways might compensate the non-canonical functions of telomerase in *tert−/−* embryos, as suggested in the context of Tert−/− mice [29], the acute inhibition of Tert in wt embryos causes the severe effects we observed in our experiments.

It is well-known that critically short telomeres elicit the activation of DDR with formation of DNA damage foci localized at telomeres, followed by the upregulation of TP53 and the induction of cyclin-dependent kinase inhibitors p21 and p16 [30]; these cells usually stop their cell cycle in G1-phase and physiologically undergo senescence or apoptosis programs, depending on cell type [30]. It has been demonstrated that long-term inhibition of telomerase activity by BIBR in dividing cells may induce telomere shortening that elicits activation of DDR at telomeres and apoptosis [31]. However, the anti-proliferative and pro-apoptotic effects associated with the activation of DDR we observed in wt zebrafish upon short-term Tert inhibition are unrelated to telomere shortening and dysfunction. Indeed, the DNA damage foci in zebrafish embryos induced by BIBR are distributed randomly in the genome, rather than specifically located at telomeres and the range of telomere length is not affected by the short-term treatment. Notably, all these effects are specifically related to Tert inhibition since BIBR treatment shows no effect in *tert−/−* embryos.

These in vivo results confirm our previous data in in vitro models [15], reinforcing the concept that telomerase has telomere length-independent effects on cell proliferation and survival and that these effects involve the induction of telomere-unrelated DNA damage. Thus, Tert per se seems to exert tumor-promoting activities that are independent from its canonical role of telomere length maintenance. Consistently with our in vivo results, a non-canonical function of telomerase was also previously described in zebrafish by Imamura et al. [17]; during knockdown experiments the authors demonstrated that Tert deficiency in zebrafish induced a defect in hematopoiesis and negatively impacted the survival of blood cells through a potent and specific effect on the gene expression of key regulators in the absence of telomere dysfunction and shortening.

Classically, a limitation of the approach based on telomerase inhibition may result from the activation of the alternative lengthening of telomeres mechanism, known as ALT, which has been described as a compensatory mechanism triggered by telomere erosion after long-term telomerase inhibition [32]. Nevertheless, the independence of the effects of BIBR treatment from telomere shortening and its efficacy soon after the beginning of the treatment, prompt re-evaluating telomerase as a promising therapeutic target.

In the light of the possible use of TERT inhibitors as antineoplastic therapy, we explored the effects of TERT inhibition by BIBR in human malignant B cells xenografted in zebrafish embryos. Using flow cytometry analysis, we were able to accurately determine the number of human tumor cells in zebrafish embryos at different time points after injection and analyze the proliferation and/or apoptotic rate, reflecting the evolution/progression of the tumor. The finding that short-term TERT inhibition negatively impacts proliferation and viability of human malignant B cells xenografted in zebrafish embryos, both in the pre- and post-transplantation treatment approach, strongly enforces the validity of this strategy to counteract tumor growth. Thus, therapeutic effectiveness of targeting TERT in antineoplastic treatment could be exploited besides its role on telomere length maintenance, circumventing the delayed therapeutic effects that are inherent to original tumor telomere length.

## 4. Materials and Methods

### 4.1. Zebrafish Lines and Maintenance

All experiments were performed in accordance with the European and Italian Legislations and with permission for animal experimentation from the Local Ethics Committee of the University of Padova (protocol numbers 569/2018-PR and 259/2020-PR). Zebrafish were maintained in a temperature-controlled (28.5 °C) environment and fed as described by Kimmel et al. [33]. The telomerase mutant line (allele *tert^hu3430^*), generated by N-Ethyl-Nitrosourea (ENU) mutagenesis, has a T-A point mutation in the tert gene that introduces an early stop codon [20]; this line was employed as negative control. The transparent casper strain (*mpv17−/− mitf−/−*) of zebrafish was employed for the xenotransplantation experiments.

A stock solution of BIBR at a concentration of 10 mM was prepared by dissolving the compound in sterile DMSO, divided into aliquots and stored at −80 °C until use. BIBR or DMSO were administered to wt and *tert−/−* zebrafish embryos in the fish water (60  µg/mL E3, 0.1 % methylene blue) at the stage of 12 hpf and samples were analyzed at 24 hpf, i.e., after 12 h of treatment, or as otherwise indicated.

### 4.2. Quantification of Telomerase Activity by Real-Time Polymerase Chain Reaction

Quantification of telomerase activity by real-time PCR method was performed as previously described [34,35]. Protein lysates were prepared from 50 BIBR treated and 50 control treated embryos at 24 hpf. Briefly, embryos were manually dechorionated, deyolked with salt solution (55 mM NaCl, 1.8 mM KCl, 1.25 mM NaHCO3), and incubated in 40 μL of 3-((3-cholamidopropyl) dimethylammonio)-1-propanesulfonate (CHAPS) buffer (0.5% CHAPS, 10 mM Tris HCl, pH 7.5, 1 mM MgCl2, 1 mM ethylene glycol-bis(β-aminoethyl ether)-N,N,N′,N′-tetraacetic acid (EGTA), 0.1 mM phenylmethyl-sulfonyl fluoride, 5 mM β-mercaptoethanol, and 10% glycerol) at 4 °C for 60 min. The lysate was then centrifuged at 12,000× *g* for 30 min at 4 °C and the supernatant was collected. The protein concentration was measured using NanoDrop spectrophotometry (ND-1000; Thermo Fisher Scientific, Wilmington, DE, USA). Telomerase activity was evaluated by a real-time PCR method, using 250 ng of protein extract for each sample. Threshold cycle values (Ct) of the samples were plotted against a standard curve generated from serial five-fold dilutions starting from 1250 ng protein extract from telomerase-positive BL41 cells. Lysates derived from telomerase-negative U2OS cells and from heat shock treatment were included in each plate. Each sample was analyzed in duplicate and values are expressed as relative units.

### 4.3. Immunohistochemistry and Immunofluorescence

Samples were manually dechorionated, fixed with 4% paraformaldehyde (PFA) in phosphate-buffered saline (PBS) overnight and then stored in 100% methanol at −20°C. Rehydrated embryos were then permeabilized in cold acetone and saturated in 0.5% Triton X-100, 1% DMSO, 1% bovine serum albumin (BSA), and 2% normal goat serum (Life Technologies, Carlsbad, CA, USA). The mitotic foci were highlighted by anti-phospho histone H3 (pHH3) antibody (pS10) (EDM Millipore, Merck, Billerica, MA, USA) followed by appropriate secondary antibody conjugated with alkaline phosphatase (EDM Millipore, Merck, Billerica, MA, USA) and visualized under a Leica M165FC dissecting microscope (Leica Microsystem, Wetzlar, Germany) and then with a Nikon C2 H600L confocal microscope (Nikon, Tokyo, Japan). S-phase was analyzed employing the anti-PCNA antibody (DAKO, Glostrup, Denmark) followed by Alexa Fluor 594 secondary antibody goat antimouse (Life Technologies, Carlsbad, CA, USA) and visualized at the fluorescence Leica M165FC dissecting microscope and then with a Nikon C2 H600L confocal microscope. All images were analyzed with ImageJ software [36].

### 4.4. TUNEL Assay

Apoptosis rate was evaluated by employing the DeadEnd™ Fluorometric TUNEL System (Promega, Madison, WI, USA). Shortly after manual dechorionation, embryos were fixed with 4% PFA and stored in 100% methanol at −20 °C. Rehydrated samples were then treated with proteinase K, incubated in 2:1 ethanol:acetic acid and then subjected to TUNEL assay, according to the manufacturer’s instructions. Apoptotic foci were visualized using the fluorescence Leica M165FC dissecting microscope and then with a Nikon C2 H600L confocal microscope. All images were analyzed with ImageJ software [36]. DeadEnd™ Fluorometric TUNEL System was also employed to detect apoptotic cells in cell suspensions from enzymatically dissociated xenograft embryos (see below) according to the manufacturer’s instructions. Apoptotic rate was quantitated by flow cytometry analyses in an LSR II cytofluorimeter (Becton-Dickinson, San Jose, CA, USA). Data were processed with FACSDiva™ Software (Becton-Dickinson, San Jose, CA, USA) and analyzed using Kaluza^®^Analyzing Software v.1.2 (Beckman Coulter, Fullerton, CA, USA).

### 4.5. Western Blotting

Protein lysates were prepared from 50 BIBR-treated and 50 control-treated embryos at 24 hpf. Briefly, embryos were manually dechorionated, deyolked with salt solution (see above), and incubated in ice-cold radioimmunoprecipitation assay (RIPA) lysis buffer (Cell Signaling Technology, Danvers, MA, USA) containing 1× Halt protease and phosphatase inhibitor cocktail (Thermo Fisher Scientific, Loughborough, UK). Proteins obtained from the supernatant after centrifugation for 30 min at 15,000× *g* were quantified using a BCA Protein Assay Kit (Pierce, Rockford, IL, USA). Equal amounts of proteins (50 μg) were loaded on 4–20% TGX gel (Bio Rad, Berkeley, CA, USA) and blotted on polyvinylidene fluoride (PVDF) membranes (Ge Healthcare Life Science, Cytiva, Marlborough, MA, USA). The expression of RNR-R2, marker of S-phase, γH2AX, marker of DNA damage, and α-tubulin (as loading control) were evaluated by anti-RNR-R2 (B-Bridge, Santa Clara, CA, USA), anti-γH2AX (pS139) (GeneTex, Alton Pkwy Irvine, CA, USA) and anti-α-tubulin (Sigma-Aldrich, St. Louis, MO, USA) antibodies, respectively. Blots were incubated with appropriate peroxidase-conjugated secondary antibody and stained with chemiluminescent detection kit (SuperSignal West Pico Chemiluminescent Substrate, Pierce). Quantitation of the signal was performed with ImageJ software [36]. The uncropped blots and densitometric analysis are shown in the Supplementary Materials (Figure S6).

### 4.6. Cell Cycle Analysis

To evaluate cell cycle profile, 50 wt and *tert−/−* embryos, treated with BIBR or DMSO, were manually dechorionated and disaggregated using small pellet pestle in 500 µL of Dulbecco’s Modified Eagle Medium (DMEM) (Sigma-Aldrich, St. Louis, MO, USA)-10% fetal bovine serum (FBS) (Gibco, Milano, Italy). Subsequently, cell suspensions were filtered through 70 μm nylon mesh and cell viability was assessed by Trypan blue exclusion in a Countess automated cell counter (Invitrogen, Life Technologies, Carlsbad, CA, USA) and was always higher than 80%. Cell suspensions were then employed for PI staining as previously described [15]. Samples were analyzed by flow cytometry and cell cycle profiles were analyzed with ModFit LT Cell Cycle Analysis software (version 2.0) (Verity software House, Topsham, ME, USA).

### 4.7. Telomere Length Measurement

The telomere length was determined by TRF analysis using the TeloTAGGG telomere length assay kit (Roche Diagnostic GmbH, Basel, Switzerland) according to the manufacturer’s instructions. Shortly, DNA was extracted with the phenol-chloroform method from dechorionated embryos; after digestion, the fragments were separated by gel electrophoresis and transferred to a nylon membrane by Southern blotting. Telomeric sequences were labeled by a specific probe, then recognized by an alkaline phosphatase conjugated antibody and visualized with a chemiluminescent substrate. In addition, relative telomere length (T/S) was estimated by quantitative real time PCR, as previously described [37], using the primer pair TEL1B and TEL2B [37] and primer pair Forward: 5′-ACCACTTCAAGCGGACAGAG-3′ and Reverse: 5′-CCGGCTTCTGATCTTTCTGC-3′ for the zebrafish single copy housekeeping *gr* gene [38]. T/S value obtained from untreated wt zebrafish embryos was employed as reference.

### 4.8. Combined FISH/Immunofluorescence

The rate of dysfunctional telomeres was evaluated by the Telomere Dysfunction-induced Foci (TIF) analysis, which measures the co-localization of telomere signals (FISH) and DNA damage foci (IF). Cellular suspensions were obtained from dechorionated and deyolked treated and mock-treated embryos by incubation with 0.25% Trypsin–EDTA for 12 min at 28 °C; these cells were cytospun onto glass slides. Telomeres were stained with the Telomere PNA FISH Kit/Cy3 (DAKO, Glostrup, Denmark) and DNA damage foci were highlighted by anti-γH2AX antibody (GeneTex, Alton Pkwy Irvine, CA, USA) followed by Alexa Fluor 488 anti-rabbit secondary antibody (Thermo Fisher Scientific, Loughborough, UK), as previously detailed [15]. Slides were mounted with 4′,6-diamidino-2-phenylindole (DAPI)/antifade solution and analyzed with fluorescence microscope, as previously described [15].

### 4.9. Cell Lines

The 4134/Late LCL expressing high endogenous levels of TERT was obtained by infecting peripheral blood mononuclear cells from normal donor with the B95.8 EBV strain. Establishment and characterization of this cell line has already been described [15,39]. BL41 is an EBV-negative Burkitt lymphoma cell line with translocated v-myc avian myelocytomatosis viral oncogene homolog *MYC* gene (kindly provided by Martin Rowe, Cancer Center, University of Birmingham, Birmingham, UK). LCL and BL41 cells were cultured in Roswell Park Memorial Institute (RPMI)-1640 medium (Euroclone, Milano, Italy), supplemented with glutamine 4 mM, 50 mg/mL gentamycin (Sigma-Aldrich, St. Louis, MO, USA) and 10% heat-inactivated FBS (Gibco, Milano, Italy) at 37 °C and 5% CO_2_. Cell lines were tested and resulted negative for mycoplasma contamination.

Before being injected into zebrafish, cells were treated for 16 h with BIBR 30 µM or DMSO as control. The cell viability was checked by Trypan blue exclusion as described above and was always equal to or above 90% for both treatments.

### 4.10. Xenotransplantation of Human Cancer B Cells in Zebrafish Embryos

Seventy-two hpf casper embryos were collected, dechorionated and soaked in fish water at 28 °C and a proper control group (non-xenografted embryos) was kept isolated. Embryos were then anaesthetized using 1.2 mM tricaine (Sigma-Aldrich, St. Louis, MO, USA) and placed in a 1.5% (*w*/*v*) agarose Petri dish to orient them in a lateral position allowing the cells transplantation directly into the yolk sac. Approximately 300 cancer cells, fluorescently labeled with CM-DiI following the manufacturer’s instructions, were loaded into a borosilicate glass capillary needle and manually injected using a microinjector attached to a micromanipulator (Leica Microsystems, Wetzlar, Germany). The number of injected cells was counted microscopically by dispensing the same volume of injected cells on glass slides using the same glass capillary needle and injection pressure. After injection, embryos were kept in fish water at 28 °C. Two hours hpx, the embryos were selected according to the intensity of engrafted mass, and non-fluorescent embryos or embryos with fluorescent cells outside the place of injection were discarded. The chosen embryos were incubated at 32 °C (a compromise between the 28 °C ideal for the zebrafish and the 37 °C optimal for human cells) and monitored daily with the fluorescent stereomicroscope M165FC (Leica Microsystem, Wetzlar, Germany) equipped with a Leica DFC7000 T digital camera. In additional experiments, embryos were xenografted with untreated tumor cells labeled with CM-DiI and, 24 hpx, tumor cells were injected with either BIBR (10 nL of 30 µM solution in PBS) or an equivalent volume of PBS containing DMSO as control.

### 4.11. Embryos Dissociation and Flow Cytometry Analysis

The dissociation of zebrafish embryos at a single cell suspension was performed as described in Bresciani et al. [40]. Briefly, about 10 euthanized embryos per condition were transferred into a 1.5 mL Eppendorf tube and washed twice with PBS. The dissociation mix, composed of 0.25% Trypsin–EDTA (Gibco, Milano, Italy) and collagenase 8 mg/mL (Sigma-Aldrich, St. Louis, MO, USA), was pre-warmed at 30 °C and added allowing the disruption of all embryos by pipetting in heat-block at 30 °C. After mechanical homogenization, fresh-made 30 °C pre-warmed DMEM (Sigma-Aldrich, St. Louis, MO, USA)-10% FBS was added to stop the enzymatic dissociation. Pelletized cells were washed with PBS, resuspended with DMEM-10% FBS and filtered through 70 μm nylon mesh. Cell viability was assessed by Trypan blue exclusion as described above and was always higher than 80%. Cell suspensions were then employed to monitor fluorescent cells for proliferation and apoptosis by flow cytometry analysis in an LSR II cytofluorimeter.

### 4.12. Statistical Analyses

Statistical analyses were performed with SPSS software version 25 (IBM, Armonk, NY, USA). Results were analyzed with *t*-test and *p*-values < 0.05 were considered significant.

## 5. Conclusions

Our results demonstrated that the short-term inhibition of Tert in zebrafish embryos triggers DDR unrelated to telomere dysfunction and compromises cell proliferation and viability, enforcing the concept that telomerase per se exerts telomere length-independent pro-tumorigenic processes. Accordingly, short-term TERT inhibition results in anti-proliferative and pro-apoptotic effects on xenografted human malignant B cells. Taken together, these data support therapeutic application of TERT inhibitors to counteract tumor growth, regardless of telomere length of cancer cells.

## Supplementary Materials

The following are available online at https://www.mdpi.com/2072-6694/12/8/2052/s1, Figure S1: Effect of short-term treatment with different doses of BIBR on zebrafish embryos viability, Figure S2: Effect of BIBR treatment on telomerase activity of zebrafish embryos, Figure S3: Short-term Tert inhibition does not affect telomere length, Figure S4: Flow cytometry analysis of cell suspensions from dissociated zebrafish embryos, Figure S5: Proliferation of BL41 cells xenografted in zebrafish embryos, Figure S6: Full-size images of uncropped blots and their densitometric quantification.
