# Supplementary material for: Anti-Proliferative and Pro-Apoptotic Effects of Short-Term Inhibition of Telomerase In Vivo and in Human Malignant B Cells Xenografted in Zebrafish
Source: Cancers (Basel). 2020 Jul 25;12(8):2052. doi: 10.3390/cancers12082052 (PMC7463531; doi:10.3390/cancers12082052)
Supplement: Supplementary file 1 [file cancers-12-02052-s001.pdf]

Supplementary Materials: Anti-Proliferative and Pro-Apoptotic Effects of Short-Term Inhibition of Telomerase In Vivo and in Human Malignant B Cells Xenografted in Zebrafish

Silvia Giunco, Manuela Zangrossi, Francesca Dal Pozzolo, Andrea Celeghin, Giovanni Ballin, Maria Raffaella Petrara, Aamir Amin, Francesco Argenton, Miguel Godinho Ferreira and Anita De Rossi

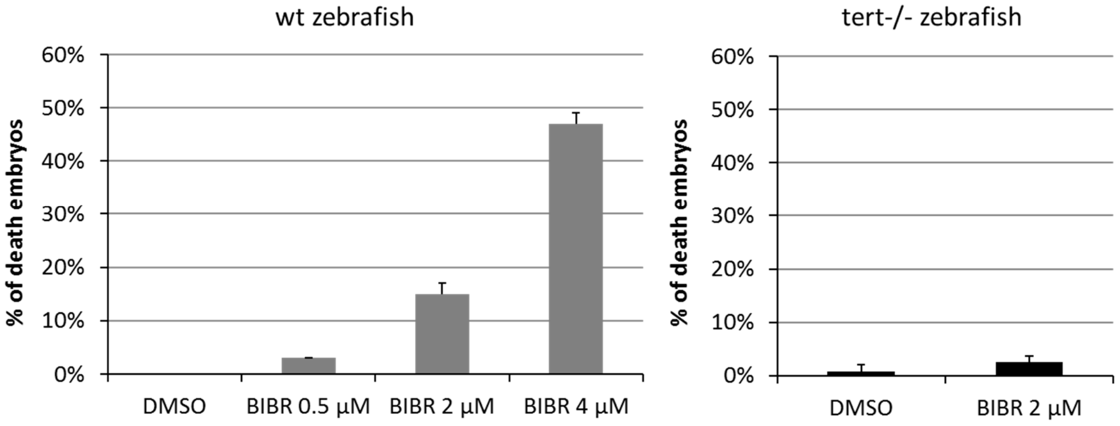

Figure S1. Effect of short-term treatment with different doses of BIBR on zebrafish embryo viability. Wild-type (wt) and *tert* mutant *tert*$^{hu3430/3430}$ (*tert*-/-) zebrafish embryos were exposed to the indicated doses of BIBR, or DMSO as control, for 12 h, from 12 to 24 h after fertilization. Values represent mean and standard deviation (SD) (bar) of percentage of death embryos from three independent experiments of 20 embryos per group.

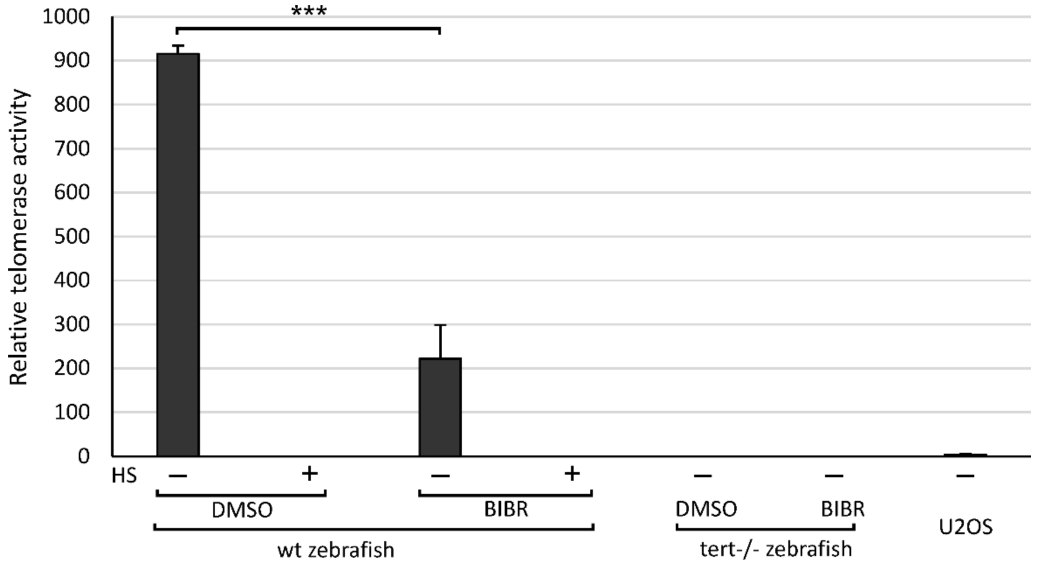

Figure S2. Effect of BIBR treatment on telomerase activity of zebrafish embryos. Both wild-type (wt) and *tert* mutant *tert*$^{hu3430/3430}$ (tert-/-) zebrafish embryos were treated with 2 µM BIBR, or DMSO as control, for 12 h, and the telomerase activity was assessed by TRAP real-time PCR, as detailed in Materials and Methods. Heat shock protein extracts (HS) and telomerase negative U2OS cells were used as negative controls. Data represent the mean and SD (bar) of telomerase activity in relative units from three separate experiments. Significant difference between values in BIBR-treated embryos *versus* DMSO-treated embryos is shown: *** $p < 0.001$.

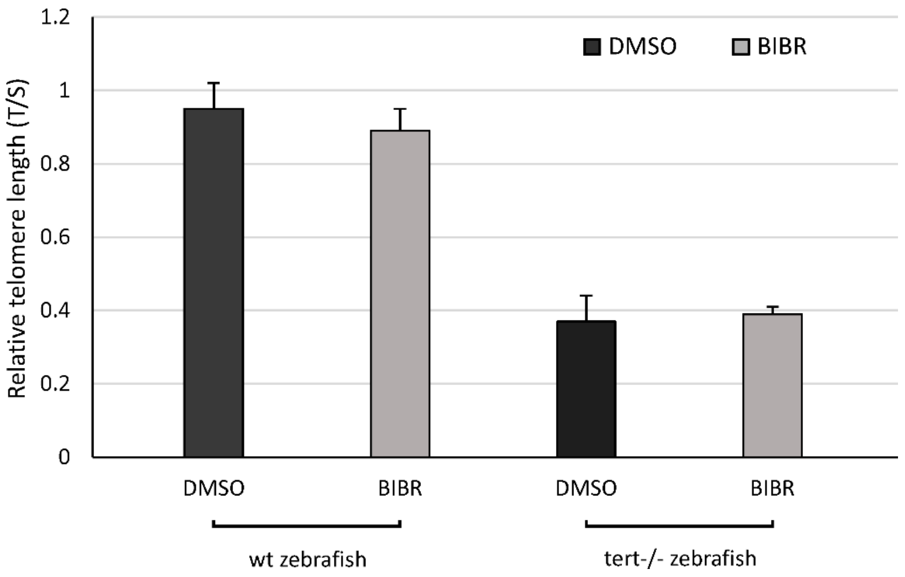

Figure S3. Short-term Tert inhibition does not affect telomere length. DNA from wild-type (wt) and *tert* mutant *tert*$^{hu3430/3430}$ (*tert-/-*) zebrafish embryos treated with BIBR or DMSO for 36 h, from 12 to 48 hpf, was employed to estimate the mean of relative telomere length (T/S) assessed by quantitative real time PCR analysis. The graph represents the T/S values and SD (bar) from three separate analyses. T/S value obtained from untreated wt zebrafish embryos was employed as reference.

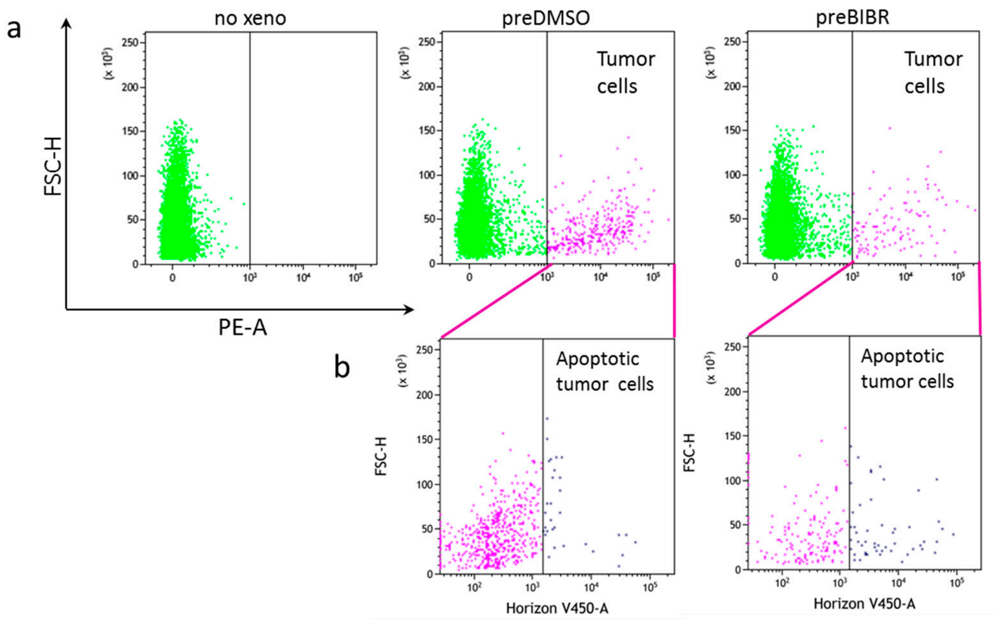

Figure S4. Flow cytometry analysis of cell suspensions from dissociated zebrafish embryos. Xenografted fluorescent 4134/Late cells, pretreated with BIBR (preBIBR) or DMSO (preDMSO), were analyzed by flow cytometry in dissociated embryos at 72 hpx. (**a**) Representative panels of flow cytometry gating strategy for selection of CM-DiI labelled human cells (PE-A); non-xenografted embryos (no xeno) were employed to set the threshold. (**b**) Cell suspensions from enzymatically dissociated embryos at 72 hpx were processed by TUNEL assay for detection of apoptotic rate (Horizon V450-A) and analyzed by flow cytometry selecting CM-DiI fluorescent population as gating in (**a**).

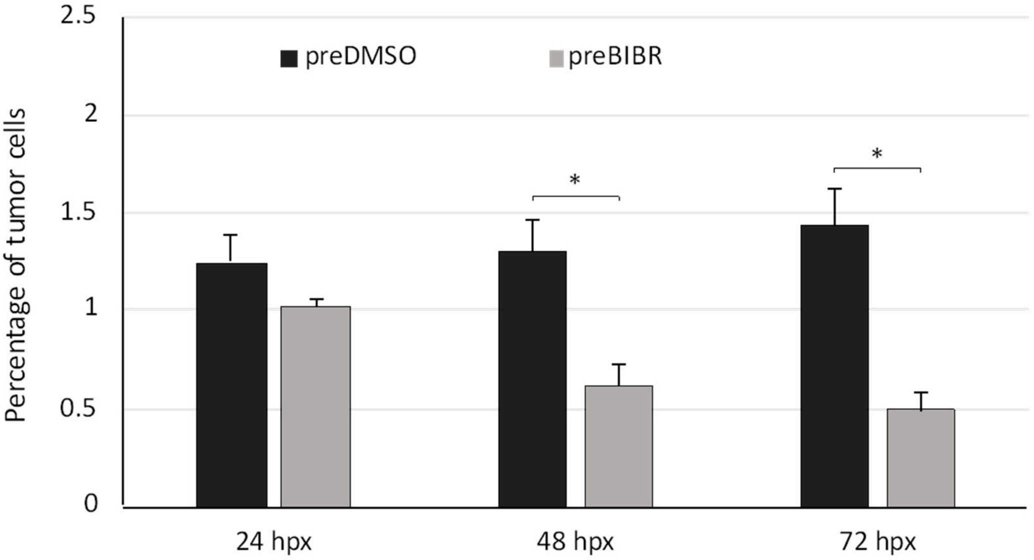

Figure S5. Proliferation of BL41 cells xenografted in zebrafish embryos. Xenografted fluorescent tumor cells, pretreated with BIBR (preBIBR) or DMSO (preDMSO), were detected in enzymatically dissociated embryos by flow cytometry. Data represent percentage of BL41 cells in zebrafish embryos according to time post-xenograft (hpx). Values are means and SD (bar) of two separate experiments of 10 embryos per group. * $p < 0.05$.

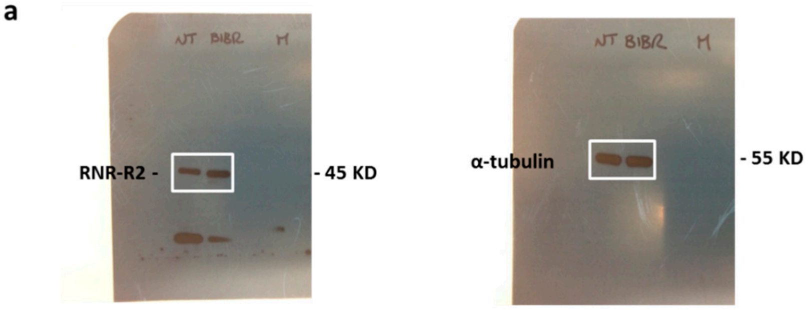

Area	R2-RNR	αTUB	Norm RNR/αTUB*100	BIBR/DMSO
Zebra 24hpf NT (DMSO)	43606	84663	51,5	1,00
Zebra 24hpf BIBR 2uM	72290	77435	93,4	1,81

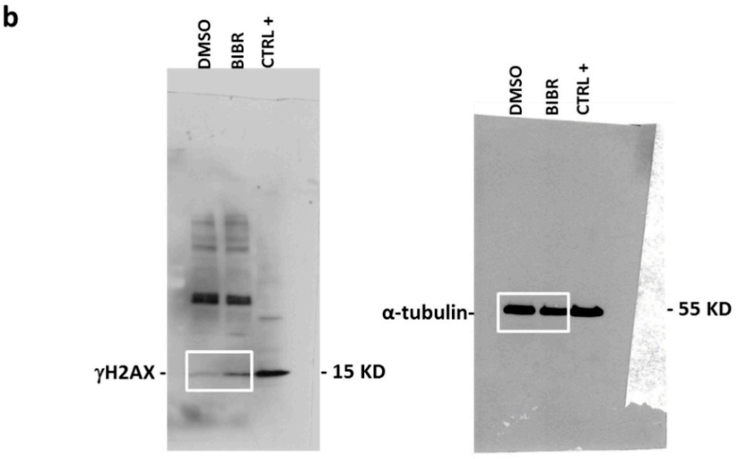

			Norm	Norm
	γH2AX	TUB	γH2AX/αTUB*100	BIBR/DMSO
DMSO	29.488.028	89.843.593	32,8	1,00
BIBR	88.295.291	71.096.200	124,2	3,78

Figure S6. Full-size images of uncropped blots and their densitometric quantification**.** (**a**) Western blot analysis of RNR-R2 and α-tubulin expression in lysates from wt zebrafish treated with BIBR or DMSO (NT) showed in Figure 1. (**b**) Western blot analysis of γH2AX and α-tubulin expression in lysates from wt zebrafish treated with BIBR or DMSO showed in Figure 2.

© 2020 by the authors. Licensee MDPI, Basel, Switzerland. This article is an open access article distributed under the terms and conditions of the Creative Commons Attribution (CC BY) license (http://creativecommons.org/licenses/by/4.0/).
